# Viruses in glioblastoma: an update on evidence and clinical trials

**DOI:** 10.1038/s44276-024-00051-z

**Published:** 2024-04-19

**Authors:** Bavani Gunasegaran, Caroline L. Ashley, Felix Marsh-Wakefield, Gilles J. Guillemin, Benjamin Heng

**Affiliations:** 1 Macquarie Medical School, Faculty of Medicine, Health and Human Sciences, Macquarie University, North Ryde, Sydney, NSW Australia; 2 Charles Perkins Centre, The University of Sydney, Camperdown, NSW Australia; 3 School of Medical Sciences Faculty of Medicine and Health, The University of Sydney, Camperdown, NSW Australia; 4 Centenary Institute, Camperdown, NSW Australia; 5 IPB University, Bogor, Indonesia

## Abstract

**Background:**

Glioblastoma (GB) is a lethal and aggressive brain tumour. While molecular characteristics of GB is studied extensively, the aetiology of GB remains uncertain. The interest in exploring viruses as a potential contributor to the development of GB stems from the notion that viruses are known to play a key role in pathogenesis of other human cancers such as cervical cancer. Nevertheless, the role of viruses in GB remains controversial.

**Methods:**

This review delves into the current body of knowledge surrounding the presence of viruses in GB as well as provide updates on clinical trials examining the potential inclusion of antiviral therapies as part of the standard of care protocol.

**Conclusions:**

The review summarises current evidences and important gaps in our knowledge related to the presence of viruses in GB.

## Glioblastoma

Glioblastoma (GB) is the most common and aggressive form of malignant primary brain tumour [1] and is considered a central nervous system (CNS) grade 4 tumour based on WHO classification [2]. GB is recognised as an isocitrate dehydrogenase (IDH)-wildtype diffuse glioma and the revised 2021 WHO classification of CNS tumours no longer includes the term ‘IDH-mutant glioblastoma.’ Instead, it is now categorised as Astrocytoma, IDH-mutant, encompassing CNS grade 2–4 tumours [2]. Majority of GB arise *de novo*, without clinical symptoms or histological evidence of a malignant lesion. They primarily affect older individuals and are genetically distinguished by specific molecular alterations, even in the absence of high-grade histopathologic features. A GB diagnosis is confirmed by the presence of at least one of the following molecular features: EGFR amplification, TERT promoter mutation, or combination gain of chromosome 7 and loss of chromosome 10 copy number alterations [3, 4]. The origin of GB within the CNS distinguishes itself from the more common secondary brain cancer known as astrocytoma, IDH mutant (CNS WHO Grade 4). The latter arises from metastasis originating in primary sites, such as breast, skin, or lung cancer [5].

## Risk factors of GB

A considerable number of GB patients do not exhibit identifiable risk factors for tumour development [6]. Currently, the sole confirmed risk factor is exposure to high-dose ionising radiation [7–9]. There is no epidemiological evidence substantiating a link between brain tumour development and the use of mobile phones [10]. Some genetic syndromes, such as neurofibromatosis, Li-Fraumeni and von Hippel-Lindau syndrome, have been demonstrated to be associated with a small percentage of GB patients [8].

While histological analyses play a crucial role in diagnosing GB, recent breakthroughs, particularly in the field of genetics, have significantly enhanced our comprehension of these tumours [11]. Among the genetic alterations observed in GB, the mutation of the IDH gene stands out as the most recognised and extensively studied. IDH enzymes, specifically IDH1 and IDH2, are homodimeric enzymes involved in the Krebs cycle, converting isocitrate to alpha-ketoglutarate [12, 13], that plays a vital role in the production of adenosine triphosphate during cellular energy generation [14].

Another noteworthy gene is O6-methylguanine-DNA methyltransferase (MGMT). MGMT serves as a DNA repair enzyme with a pivotal role in conferring chemoresistance to alkylating agents. It encodes a DNA repair protein of the same name responsible for removing alkyl groups from the O6 position of guanine, a crucial site for DNA alkylation. Chemotherapy-induced alkylation triggers apoptosis and cytotoxicity at this location. The overexpression of MGMT in tumour cells has the potential to hinder the therapeutic effects of alkylating drugs [15]. In more than 40% of GB patients, MGMT undergoes epigenetic inactivation through hypermethylation [16]. For detailed information on other genetic alterations in GB, we direct the attention of those interested to recent articles [17–20].

## Treatment of glioblastoma

GB is associated with a grim prognosis, marked by exceedingly high morbidity and mortality rates. The median overall survival (OS) for GB patients ranges from 12 to 15 months, with a 5-year survival rate of less than 16% in children and 5% in adults [21–23]. Prognosis is notably poorer for elderly patients aged 65 or older, who face an average survival of 8.5 months from diagnosis [24]. The current standard of care treatments for GB includes a combination of maximal surgical resection of the tumour mass followed by radiotherapy and chemotherapy [25].

Currently, the Food and Drug Administration has approved only two drugs for the treatment of GB: Temozolomide, an alkylating chemotherapeutic agent and Bevacizumab, a monoclonal antibody that targets and inhibits vascular endothelial growth factor. These medications are employed in the treatment of newly diagnosed GB and recurrent GB, respectively [26]. Additionally, tumour-treating fields therapy, also known as alternating electric field therapy, involves the use of low-intensity, intermediate-frequency electric fields to the scalp to disrupt the division of rapidly growing cells. Clinical trials of tumour-treating fields have reported enhancements in overall long-term survival in patients with primary or recurrent GB [27, 28].

Emerging therapeutic strategies encompass targeted therapies, honing in on specific molecular markers within tumours and immunotherapies such as CAR-T cell therapy and immune checkpoint inhibitors, which aim to leverage the immune system in combating cancer. The exploration of personalised medicine and genomics seeks to tailor treatments to individual patients, while innovative drug delivery methods like convection-enhanced delivery enhance drug effectiveness [29]. These diverse approaches signal a shift towards more precise, personalised and potentially less toxic treatments, although further research and clinical trials are needed to establish their efficacy and safety. This review focuses on the recent evidence of viral involvement in GB, with a focus on clinical trials in the field of anti-viral drugs. We direct the attention of those interested to recent reviews [29–31] that cover current and new approaches to treat the GB.

The lack of novel treatments for GB is not for lack of effort. As of October 31, 2023, there are currently 1453 completed and ongoing trials registered under ‘glioblastoma’ on ClinicalTrials.gov (excluding trials that are suspended, terminated, withdrawn, or of unknown status). The resistance of GB to treatment can be attributed to various distinctive characteristics of the tumour, contributing to the absence of substantial advancements in GB treatment over the past decade.

## Development of GB and its immunosuppressed characteristics

While inflammatory pathways are present in GB, they increase the chances of reactive species involvement in mutagenesis, while concurrently attracting macrophages and microglia into the local tumour environment. GB has a highly ineffective host anti-tumour response, despite attracting macrophages and microglia into the local environment [32, 33]. There are several factors that contribute to the ineffective response [32–34]. There is a reduced expression of major histocompatibility complex (MHC), also often referred to as the human leucocyte antigen system in humans, consisting of class I and class II molecules, within the tumour [35, 36]. Consequently, this reduces the ability to present neoantigens to the adaptive immune cells. When MHC is downregulated, GB cells upregulate immunomodulatory surface ligands such as Programmed death-ligand 1 that promotes T cell exhaustion and anergy [37].

Additionally, there are elevated immunomodulatory biochemical pathway enzymes such as indole 2,3-dioxygenase and tryptophan 2,3-dixoygenase of the kynurenine pathway that contributes to immune suppression [38]. The tumour microenvironment of GB has high levels of inflammatory nitric oxide synthase expression, alongside an elevated reactive nitrogen and oxygen environment that can promote angiogenesis, cell proliferation and migration/invasion [39, 40]. High levels of cyclooxygenase-2 were also expressed in GB [41, 42]. An increased cyclooxygenase-2 expression is often associated with inflammatory processes in response to tissue damage, tumourigenesis [42] and other stimuli such as growth factors, lipopolysaccharides and cytokines [43].

While infiltrating T cells might be present, a third of the cells within the tumour exhibit macrophage markers. Therefore, it is probable that these macrophages are a mix of intrinsic microglial cells from the CNS and cells derived from infiltrating monocytes [32, 44]. The effector T cells found in GB are non-functional. The inactivation of infiltrating T cells might be attributed to T cell anergy, which can result from the influence of tumour-associated immature dendritic cells that render T cells inactive [32]. Regulatory T cells are found in high levels in GB that also contribute to the inhibition of T cell proliferation and activation [45]. Despite evidence suggesting an increased level of microglia/macrophage infiltration and inflammation, GB patients are also known to harbour systemic defects in cell-mediated immunity as well as cytotoxic T cell functions. GB is known to exist in a highly immunosuppressive environment, marked by the elevated expression levels of immunosuppressive factors like the transforming growth factor β and interleukin-10 [46–48].

This paradoxical inflamed and immunosuppressed tumour microenvironment not only allows the tumour to grow undetected and aggressively, but this also creates a window of opportunity for pathogenic agents to evade immune detection and infect the tumour site [49]. Indeed, there are several solid tumours with a suppressed immune system that are known to be associated with oncogenic viral infections [49, 50].

## Role of infectious pathogens in human cancers and possible viral associations in GB

In 2018, approximately 13% of all new cancer cases were attributed to infections that involved viruses, bacteria and schistosomes [51, 52]. The top infectious agents noted were *Helicobacter pylori* (approximately 37%, predominately non-cardia gastric adenocarcinoma), human papillomavirus (HPV; approximately 31%, predominately cervical carcinoma), hepatitis B virus (HBV; approximately 16%, hepatocellular carcinoma) and hepatitis C virus (HCV; approximately 7.3%, hepatocellular carcinoma). The remaining cases were associated with Epstein-Barr virus (EBV), human T-lymphotropic virus (HTLV), Merkel cell polyomavirus (MCPyV) and Kaposi’s sarcoma-associated herpesvirus/Human herpesvirus type 8 (KSHV/HHV-8) [51]. Approximately 20% of all human cancers worldwide are caused by viruses, with seven viruses causally linked to human cancers: HBV, HCV, HPV, EBV, HTLV, MCPyV and KSHV [53–55]. Chronic viral hepatitis such as that caused by HBV and HCV can lead to the onset of liver cancer [56, 57]. HPV are known causes of cervical, anal, penile and oropharyngeal cancer [58]. EBV was the first virus to be shown to cause cancer in humans and has been associated with a wide range of human cancers deriving from mesenchymal cells, epithelial cells and lymphocytes [59]. EBV infection is associated with Burkitt’s lymphoma, B lymphoproliferative disorder, Hodgkin’s lymphoma and nasopharyngeal carcinoma [60]. HTLV was the first human retrovirus to be discovered [61]. HTLV infection almost always results in adult T cell leukaemia/lymphoma [62]. MCPyV infection can lead to the rarest form of skin cancer known as Merkel cell carcinoma [63]. KSHV is the most common infection leading to Kaposi’s sarcoma [64]. Of the tumour-associated viruses mentioned above, polyomaviruses (PyVs) and human herpesviruses (HHVs) can be neurotropic, are capable of crossing the blood-brain-barrier [65] and may contribute to neurodegenerative diseases [66], neurological brain tumours [67] and neuroinflammation [68]. However, whether viruses from these families play a causative role in GB has yet to be confirmed.

## Polyomaviruses (PyVs)

PyVs are double-stranded deoxyribonucleic acid (DNA) viruses with a small, non-enveloped icosahedral capsid that contains a circular genome. The genome comprises of three regions: control, early and late. The control region contains the origin of replication and the promoters that regulate the expression of early and late genes. Neither protein nor functional ribonucleic acid (RNA) is encoded in this region, but it is involved in viral life cycle regulation. Early and late refer to the stage in the cascade of viral gene expression when proteins from these regions are produced. The early coding region encodes both large tumour (LT) and small tumour (ST) antigen, while the late coding region encodes for viral structural proteins referred to as VP1, VP2 and VP3 [69] and/or a small accessory protein known as Agno [70]. The T antigens were so named because, historically, the PyVs John Cunningham virus (JCV), BK virus (BKV) and Simian virus 40 (SV40) were used to transform cell lines from their non-native hosts [71]. Similarly, inoculation with JCV, BHV and SV40 can induce brain tumours in animals [72]. In humans, JCV and BKV have been associated with cancers [73], however, to date only one PyV, MCPyV, has been shown to be directly tumourgenic in humans [74–76]. MCPyV causes the skin cancer Merkel cell carcinoma by integrating it’s genome into the human receptor tyrosine phosphatase type G gene (*PTPRG*) resulting in the production of MCPyV ST and LT antigen-PTPRG fusion transcripts [77]. In the context of GB, JCV, BKV and SV40 have been detected and, given their ability to induce brain tumours in animals, further investigation is warranted. All studies examining the role of PyVs in GB are summarised in Table 1.

**Table 1:** Polyomaviruses.

Year	Sample	Assay	Viral target type	Outcome	Reference
JCV					
2000	5 FFPE GB tissues	nPCR and IHC	DNA and protein (LT- antigen)	None of the samples were detected	Caldarelli-Stefano et al. [102]
2001	21 GB FFPE tissues	IHC	DNA and protein (T- antigen and VP-1)	12/21 samples were positive for early gene product (T-antigen), with no expression of VP-1 (late proteins)	Del Valle et al. [99]
2003	13 fresh GB tumours	IHC	DNA and protein (T- antigen and VP-1)	7/13 samples were positive for JCV LT 5/7 JCV Lt positive sample were also positive for VP-1	Boldorini et al. [100]
2005	21 biopsy, 17 peripheral blood and 11 cerebrospinal fluids from GB patients	PCR	DNA (LT-antigen)	11/21, 1/17 and 2/11 of samples were positive for DNA in biopsy peripheral blood, cerebrospinal fluid respectively	Delbue et al. [101]
2005	102 GB and 19 human epilepsy control brain FFPE tissues	PCR	JCV DNA	2/102 samples were positive for JCV DNA, while control brains were not	Rollison et al. [129]
2020	82 GB FFPE tissues	PCR, IHC	JCV DNA (T-antigen, transcription regularity region and VP1 gene)	3/82 tissues were positive for JCV	Limam et al. [124]
BKV					
1987	18 fresh frozen GB tissues and 5 post-mortem control brains	Dot blot hybridisation, indirect immunofluorescence, ELISA	RNA, DNA and BKV T antigen	9/18 (50%) samples were positive for BKV DNA, while none were detected in normal control tissues. 4/4 were positive for BKV T antigen, DNA and RNA	Corallini et al. [106]
1995	17 GB, 13 healthy brains and 35 whole blood samples from healthy individuals	PCR and RT-PCR	DNA (BKV LT-antigen)	BKV T antigen sequences in 15/17 (88%) GB tumour samples, all 13 (100%) healthy brain tissues and 25/35 (71.4%) whole blood samples from healthy individuals	De Mattei et al. [110]
1996	30 FFPE GB tissues and 13 normal brain tissues	PCR, indirect immunofluorescence	DNA (BKV LT-antigen)	BKV DNA in 28/30 (93%) GB tumours and 13/13 (100%) normal control brain tissues.	Martini et al. [125]
1999	28 FFPE GB tissues	PCR	DNA (BKV LT-antigen)	1/28 were positive for BKV	Huang et al. [128]
2005	102 GB and 19 human epilepsy control brain FFPE tissues	PCR	BKV DNA	3/102 GB samples were positive for BKV DNA, while control brains were not	Rollison et al. [129]
SV40					
1996	30 FFPE GB tissues and 13 normal brain tissues	PCR, RT-PCR and indirect immunofluorescence	DNA (SV40 LT-antigen)	SV40 LT antigen coding sequences in 10/30 but not in 13 control brain tissues	Martini et al. [125]
1997	4 FFPE tissues	PCR and southern blot	DNA (SV40 T-antigen)	1/4 were positive for SV40	Suzuki et al. [115]
1999	28 FFPE GB tissues	PCR	DNA (SV40 LT-antigen)	7/28 were positive. Out of the seven GB tumour biopsy that tested positive for SV40 LT antigen, adjacent normal brain tissue on the same histologic section were not positive for SV40.	Huang et al. [128]
1999	8 fresh GB tumours and 8 normal post autopsy brain tissues	Immunoprecipitation by silver staining and western blot	Protein (p53 and Tag-pRb)	SV40 T antigen was detected in 4/8 tumours, while no control brains were positive.	Zhen et al. [116]
2001	32 primary tissues	PCR	SV40 DNA (Transcription regularity region)	3/32 GB tumours contained SV40 DNA sequences but not SV40 T antigen	Kouhata et al. [117]
2002	16 fresh frozen GB tissues	PCR	SV40 DNA (NH2, COOH, T-antigen, VP1 and Transcription regularity region)	6/16 tissues were positive for SV40 T antigen, while the T antigen middle region was not detected in any samples. 0/6 samples were positive for SV40 VP1, while 4/6 samples were detected to contain the SV40 regulatory region	Martini et al. [118]
2005	20 FFPE GB samples	IHC	Protein (T-antigen)	0/20 tissues were positive for SV40 T antigens.	Sabatier et al. [119]
2020	82 FFPE tissues	PCR, ISH and IHC	SV40 DNA (T-antigen, transcription regularity region and VP1 gene)	12/82 tissues were positive for SV40	Limam et al. [124]
MCPyV					
2020	82 FFPE tissues	PCR, ISH, IHC	MCPyV DNA (T-antigen, transcription regularity region and VP1 gene)	26/82 tissues were positive for MCPyV	Limam et al. [124]

### JCV

JCV has been reported to be the causal agent of progressive multifocal leukoencephalopathy [78], a lethal disease of the CNS. Primary JCV infection is alleged to occur in the tonsils [79–81], following which the virus spreads to the epithelium of the kidney [82, 83] where it establishes a life-long, persistent infection [84]. JCV infection is typically subclinical and localised in the kidney in immunocompetent individuals. Cells permissive for JCV infection include those in peripheral blood and B cells in the bone marrow [85–90], hinting that viral spread after primary replication in the tonsil could occur by a hematogenous route. In the CNS, JCV infects astrocytes and myelin-producing oligodendrocytes [91, 92]. JCV remains dormant in healthy individuals and shown to only reactivate and cause disease in severely immunocompromised individuals [93]. While JCV has been known to cause various brain tumours in non-human primates and rodents, its direct link to human cancer is not yet fully understood [94–98].

Del Valle et al. reported that 12/21 (57.1%) of GB samples contained the early gene sequence of JCV by gene amplification, which prompted the examination of the early gene product tumour antigen by immunohistochemistry (IHC), revealing tumour antigen positive nuclei. IHC against VP1 displayed no evidence of expression of the viral late proteins in any of the specimens [99]. A similar percentage of positive changes was also reported by Boldorini et al. who detected JCV LT region in 7/13 (53.8%) fresh GB tumour specimens [100]. In another study led by Delbue et al., JCV DNA was detected in 11/21 (52.4%), 1/17 (5.9%) and 2/11 (18.2%) DNA extracted from biopsy, peripheral blood and cerebrospinal fluid (CSF) respectively from GB patients using polymerase chain reaction (PCR) [101]. Conversely, Caldarelli-Stefano et al. did not detect any JCV DNA and LT antigen in their 5 formalin-fixed paraffin-embedded (FFPE) GB tissues using nested PCR (nPCR) and IHC. The authors believed that the absence could be due to the quality of FFPE fixed tissue and the small sample size [102].

### BKV

BKV is the causative agent in BKV nephritis in renal transplant patients. BKV remains latent in the urogenital tract and is most likely transported from the donor to the recipient’s kidney [103]. BKV interacts with gangliosides for cell attachment and utilises an unidentified endocytic pathway for viral entry. While BKV can establish productive infections in natural hosts and potentially integrate into the host genome in non-permissive hosts, its direct link to cancer development remains uncertain [104]. Multiple reports have shown conflicting results regarding the presence of BKV sequences and proteins in various tumour types, specifying a lack of clear association [105]. BKV infection can be detected by using antibodies against tumour antigens and/or detection of viral DNA using PCR [103].

The first study to demonstrate the involvement of BKV in GB was led by Corallini et al. They detected BKV DNA in 9/18 (50%) of GB tumours analysed, while none were detected in normal control tissues. Due to limited material, only 4 GB tissues were tested and found positive for BKV T antigen, DNA and RNA [106]. A later study by Mattei et al. also detected BKV T antigen sequences in 15/17 (88%) GB tumour samples, all 13 (100%) healthy brain tissues and 25/35 (71.4%) whole blood samples from healthy individuals using PCR amplification and Southern-blot hybridisation. The authors further investigated these samples for expression of BKV early region by performing RT-PCR to confirm the specificity of BKV sequences in the amplified products by DNA sequence analysis. DNA sequencing on two representative BKV positive GB samples revealed that the amplified PCR products were indeed identical to the early region sequence of wild type BKV. The authors further argued that their data, along with previous studies, demonstrate that normal brain tissue [107, 108] and peripheral blood cells [109] are sites of latent BKV infection. Peripheral blood cells might transport the virus to different tissues and may be responsible for the extensive diffusion of BKV infection. This seems to be a universal feature of polyomaviruses, since JCV also infects peripheral blood cells where it remains latent [110].

### SV40

SV40 is a small DNA virus of monkey origin that can cause tumours in animals, but it mostly remains latent [111]. SV40 was transmitted to humans mainly through contaminated polio vaccines manufactured between 1955 and 1961 [112]. Several studies have reported detection of varying prevalence of serum antibodies to SV40 in study populations [113, 114]. Suzuki et al. detected the presence of SV40 T antigen in 1/4 (25%) FFPE GB tissue but not within normal control brain tissues using PCR and southern blotting. DNA sequencing of the SV40 antigen positive tissue further showed similar sequences to that of the wild-type SV40 T antigen [115]. This observation was further supported by Zhen et al. who also detected SV40 LT antigen in 4/8 (50%) fresh GB tumours using immunoprecipitation by silver staining and western blot, while all 8 normal post autopsy brain tissues were negative for LT antigen. The detection of SV40 LT antigen not only demonstrated the presence of SV40 in human brain tumours, but also suggested that the virus was biologically active to translate its viral capsid protein. The authors also noted that the SV40 LT antigen formed specific complexes with tumour suppressors p53 and retinoblastoma (Rb) protein in tumour cells. The presence of these complexes strengthens the role of SV40 in human brain tumourigenesis [116]. In a later study by Kouhata et al., they revealed 3/32 (9.4%) primary GB tumours contained SV40 DNA sequences but not SV40 T antigen. The study suggests that SV40 may have been latent in GB patients [117].

Martini et al. investigated the presence of SV40 sequences from varying regions of the SV40 genome in a variety of brain tissues. The authors utilised PCR to detect SV40 T antigen sequences in 6/16 (37%) GB tissues, while the T antigen middle region was not detected in any of the six positive samples. Positive samples were further explored by PCR for SV40 VP1 coding sequences in the late coding region and regulatory region. VP1 sequences were not observed in any samples, while 4/6 (67%) of the samples were detected to contain the SV40 regulatory region [118]. Only one study, Sabatier et al., did not detect SV40 LT antigens in 20 GB tissues via IHC [119].

### MCPyV

MCPyV is a small DNA virus with a double-stranded circular DNA [120] that is frequently found in healthy human skin, indicating that its infection is common among the general population [121]. Further research has proved that MCPyV exposure occurs in more than 80% of the general population [75, 122]. MCPyV becomes a permanent part of the skin microbiota once acquired, persisting for the rest of an individual’s life [123].

In a study performed by Limam et al., 26/82 (31.7%) of the GB FFPE tissues were positive for MCPyV DNA sequences for LT antigen using PCR and DNA sequencing. The authors found MCPyV DNA was significantly related to the presence of SV40 DNA in GB tissues. However, since MCPyV is shed from this skin it is possible that the presence of MCPyV DNA represents contamination from the skin during tissue collection rather than true infection. While the presence of MCPyV DNA significantly correlated with patient age and tumour recurrence, no further significant correlations were identified [124].

## PyV coinfection studies

There are also studies that report multiple PyVs in GB tissues. Martini et al. detected SV40 LT antigen coding sequences in 10/30 (33%) GB samples but not in the 13 healthy control brain tissues using southern blotting and hybridisation. The authors also detected BKV DNA in 28/30 (93%) of GB tumours and 13/13 (100%) of normal control brain tissues. All tumour patients were of an age that would exclude them from the period in which SV40 contaminated polio vaccines were administered. The authors also performed DNA sequence analysis of the amplified DNA products to assess SV40 specificity in 1/10 SV40 positive GB samples. While viral DNA sequences were detected to correspond to SV40 LT coding region, they also showed 2 distinct point mutations: C → T and A →G transitions at codons 110 and 115, respectively. The authors were unable to comment if the SV40 sequences detected in GB maintained their functional properties [125], as the mutated LT in the amino-terminal region of SV40 have perhaps lost their ability to transform [126, 127].

In another study of multi-viral screening in GB, Huang et al. revealed 7/28 (25%) samples to be positive for SV40 LT antigen sequences, while 1/28 (4%) were positive for BKV LT antigen in GB samples. JCV LT antigen was not detected in the cohort. Of the seven GB tumour biopsies that tested positive for SV40 LT antigen, adjacent normal brain tissue on the same histology section was not positive for SV40. Additionally, DNA sequencing was carried out on one representative GB tumour sample that was positive for SV40 LT antigen to further confirm the PCR products, which was found to be identical to the wild-type SV40 LT antigen sequence. Despite the identical sequence results, the authors cautioned that the result was inconclusive due to the small region of the LT antigen that was screened [128].

Next, Rollison et al. assessed the variability of viral detection assays for BKV, JCV and SV40 between two independent laboratories: National Institute of Neurological Disorders and Stroke (NINDS) and Johns Hopkins University (JHU). Different DNA extraction and PCR protocols with the same series of 102 GB tumour tissues were analysed. The authors noted that 2/102 (2%) and 3/102 (3%) samples were found to be positive for JCV and BKV DNA respectively in the NINDS cohort, while no samples were positive for both viruses in the JHU cohort and SV40 was not detected in either laboratory. The authors suggest BKV, JCV and SV40 were not present in most GB tumours, while a small subgroup of tumours may contain very low levels of PyVs sequence [129].

Finally in a study led by Limam et al., SV40 and JCV DNA sequences were found to be present in 12/82 (14.6%) and 3/82 (3.65) respectively, while no viral DNA of BKV were detected [124].

In conclusion, while the role of PyVs in GB remains an area of active investigation, ongoing research holds the promise of unravelling their potential contributions to the aetiology and therapeutic strategies in GB.

## Herpesviruses

Herpesviruses can be categorised into three main groups: alpha (α), beta (β) and gamma (γ). Their classification depends on their genetic organisation, replication strategies and host range [130]. Varicella-zoster virus (VZV) and herpes simplex virus (HSV) types 1 and 2 are α-herpesviruses, human herpesvirus (HHV) 6 (variants A and B) and 7 and human cytomegalovirus (HCMV) are β-herpesviruses, whilst HHV 8 and EBV are γ-herpesviruses [130, 131]. Almost all of the human population will be infected by one or more herpesviruses in their lifespan [132, 133] and typically herpesviruses can persist in the host as latent infections for an extended period after primary infection [134]. However, these latent infections may be reactivated in healthy immunocompetent individuals to cause severe disease and mortality [135]. Most herpesviruses can be neurotropic and cause severe encephalitis in immunocompetent and immunocompromised patients alike. All studies examining the role of herpesviruses in GB are summarised in Table 2.

**Table 2:** Herpesviruses.

Year	Sample	Assay	Viral target type	Outcome	Reference
EBV					
2001	Blood samples from 57 GB and 165 healthy controls	ELISA	Antibody (IgG)	48/56 GB patients were seropositive for EBV	Wrensch et al. [198]
2013	Annotated RNA-Seq data from 168 GB samples	RNA-Seq	EBV transcripts	0/168 samples were found positive for transcribed EBV viral elements	Khoury et al. [155]
2014	19 GB FFPE samples	NGS	*Unmapped sequences were analysed using a custom bioinformatics pipeline (VirusSeeker)*	EBV DNA were detected in 3/19 samples. No EBV RNA transcript was found with RNA ISH.	Cimino et al. [157]
2015	31 FFPE and 8 frozen GB tumours	qPCR	EBV gene (*LMP-1)*	Failed to detect EBV in any samples	Hashida et al. [156]
2015	18 fresh frozen primary GB specimens, but only 11 had DNA extracted	PCR, sequencing	EBV DNA (BamM region) EBV gene (*LMP-1)*	1/11 specimens to be positive for EBV DNA	Fonseca et al. [148]
2016	1 fresh frozen GB, 2GB RNA samples, RNA-seq datasets from 157 primary GB tumours and 5 normal brain and WGS datasets from 51 match blood, primary GB tumours and 20 normal blood samples	qPCR, Meta-transcriptomic and virome analysis	EBV gene (*EBER1)*	Detected no virus associations in RNA-seq datasets. Low levels of EBV DNA presence in 9/51 primary GB, 6/9 matched blood and 4/20 (20%) normal blood samples.	Strong et al. [154]
2016	19 FFPE, 20 OCT, 6 fresh frozen GB tissues 32 fresh frozen and 17 FFPE non-neurological disease control brain specimens	Multiplex digital droplet PCR assay	EBV gene (*LMP-1)*	EBV *LMP-1* sequence in 4/45 of GB samples and not in any controls. All four samples that were found to be EBV positive were FFPE samples	Lin et al. [149]
2017	21 FFPE GB sections	IHC	Protein (*LMP-1)*, EBV gene *(EBER*)	6/21 samples were positive for *LMP1* and *EBER*	Zavala-Vega et al. [203]
2017	9 tumour biopsies and 24 surgical tumour resection samples from GB patients	PCR	EBV gene (*EBNA)*	detected EBV *EBNA* sequence in 3/33	Strojnik et al. [150]
2019	82 GB FFPE tissues	PCR, IHC and ISH	EBV DNA (BamM region), Protein (*LMP-1), G*ene *(EBER*)	24/82 tissues tested EBV positive. IHC and ISH revealed 4/24 positive for *LMP-1 and EBER*	Limam et al. [151]
HCMV					
2001	Blood samples from 57 GB and 165 healthy controls	ELISA	Antibody (IgG)	37/56 GB patients were seropositive for HCMV	Wrensch et al. [198]
2002	22 GB, 9 nervous system diseases, 5 normal brain FFPE tissues	IHC, serological assay	DNA and protein *(IE1-72* and *pp65*)	8/8 GB samples were positive for HCMV DNA and proteins.	Cobbs et al. [167]
2005	8 GB tumour and 4 normal brain FFPE tissues	PCR, ISH and IHC	DNA (*pp65* and *pp150*), protein (*p52 and pp65*)	No viral DNA or proteins were detected in any of the samples	Lau et al. [181]
2006	23 fresh frozen GB tissues	PCR, IHC, ELISA	DNA (gB and IE1-72), protein (pp65, IE, EA), antibodies (anti-HCMV IgM and IgG)	No viral DNA or proteins were detected in any of the samples	Poltermann et al. [182]
2008	45 GB FFPE samples and blood samples from 20 GB patients and 17 healthy volunteers.	IHC, ISH, PCR	Protein (pp28 and gB), DNA (E1-72 and gB).	42/45 tested positive for IE1-72 protein. 30/33 IE1-72 positive samples were also positive for pp65 protein. 8/34 positive for IE1-72 DNA, 21/34 positive for gB DNA. Selected 16/16 FFPE and 16/20 whole blood were positive for HCMV DNA	Mitchell et al. [180]
2008	21 FFPE GB tissues	IHC and ISH	DNA (IE1-72), Protein (IE1-72)	IE1-72 protein and HCMV DNA was detected in all tissues.	Scheurer et al. [173]
2011	49 primary GB FFPE tissues	IHC	Protein (pp65 and IE1-72)	8/49 were positive for IE1-72 and 25/49 were positive for pp65	Lucas et al. [174]
2012	18 fresh frozen GB and 6 non-glial tumours	PCR	DNA (pp65)	6/18 of GB samples were HCMV positive. All non-glial specimens were HCMV negative	Fonseca et al. [175]
2014	16 GB and 4 non-tumour brain fresh frozen tissues	qPCR	commercial CMV detection kit	Only 12/16 GB tumours tested positive for HCMV	Ahani et al. [176]
2014	Matched 22 fresh tumour tissue and peripheral blood of GB patients	nPCR, qPCR	DNA (gB, pp65)	gB was detected in 20/22 tumours and 11/20 blood samples. 21/22 tumours and 9/20 blood samples were positive for pp65.	dos Santos et al. [177]
2014	19 matched fresh GB tumour and peripheral bloods and 6 control brain tissues	IHC, nPCR	DNA (UL55), Protein (IE1-72 and pp65)	16/19 and 12/19 of GB tissues were positive for IE1-72 and UL55 respectively. Of the 16 IE1-72 positive samples, 15/16 tested positive for pp65. 7/19 blood tested positive for HCMV DNA. No HCMV DNA and protein was detected in the control brain tissues.	Ding et al. [178]
2014	40 Frozen 19 FFPE GB tissues	qPCR, IHC and Fluorescence in-situ hybridisation	DNA (gB, IE) and protein (gB, IE)	No viral DNA or proteins were detected in any of the samples	Yamashita et al. [183]
2015	34 fresh tumour tissue	WGS	DNA	1/34 tissue tested positive for HCMV.	Tang et al. [184]
2015	31 FFPE and 8 frozen GB tumours	qPCR	DNA (IE, gB)	No HCMV were detected in these samples	Hashida et al. [156]
2017	158 FFPE and 19 Fresh GB tumours, with 119 match blood samples 48 GB patients and 91 healthy donors’ plasma	Serology, qPCR and IHC	DNA (pp65 and IE-1) and protein (IE-1), Serology (HMCV IgG)	26/158 FFPE tumours and matching 12/119 were tested positive for pp65 DNA. 65/172 FFPE and frozen tumours and matching 18/130 blood were tested positive for IE1-72 DNA. 40/158 FFPE tumours were positive for IE-1 protein. 38/48 patients were IgG positive compared to healthy controls.	Bahador et al. [179]
HHV6					
1999	18 FFPE tissues	nPCR	HHV6 DNA	1/18 tissues were positive	Chan et al. [192]
2001	31 fresh frozen primary GB tissues and 31 normal brain tissues	PCR, IHC	HHV6A/B DNA, protein (p41/38)	14/31 samples were positive. Out of which 13/14 were positive for HHV6A and 1 was HHV6B.	Cuomo et al. [193]
2012	14 GB tissues and 13 normal brain tissues	nPCR, Immunofluorescence, IHC	HHV6 DNA, protein	7/14 were positive while only 1/13 of normal brain tissues was positive. IHC revealed 5/14 were positive for HHV6 cytoplasmic and nuclear staining.	Chi et al. [194]
2016	19 FFPE, 20 OCT, 6 fresh frozen GB tissues 32 fresh frozen and 17 FFPE non-neurological disease control brain specimens	Multiplex digital droplet PCR assay	HHV6A/B DNA (U57*)*	3/19 FFPE GB samples, 3/20 frozen GB samples and 2/49 control brain tissues were positive for HHV6B	Lin et al. [149]
VZV					
1997	1) Interviews (281 GB and 443 healthy controls) 2) Blood samples (187 patients with glioma and 169 healthy controls	ELISA and interviews	Antibody (IgG)	1) Interviews: (267/381 patients with glioma and 348/414 healthy controls were tested positive 2) ELISA: 138/187 patients with glioma and 141/169 healthy controls were tested positive	Wrensch et al. [201]
1997	Telephone interviews with 281 GB and 443 healthy controls	Interviews	Medical history	Higher proportion of healthy controls (84.1%) reported to have history of chickenpox as compared to patients (70.1%)	Wrensch et al. [202]
2001	Blood samples from 57 GB and 165 healthy controls	ELISA	Antibody (IgG)	46/57 GB patients and 149/162 controls were positive for VZV	Wrensch et al. [198]
2005	Blood samples from 115 GB patients and 289 controls	ELISA	Antibody (IgG)	109/115 GB patients and 282/289 controls tested positive for VZV IgG	Wrensch et al. [199]
2011	Blood samples from 61 GB patients and 393 controls	ELISA	Antibody (IgG)	59/61 GB patients and 389/393 controls were positive for VZV	Sjostrom et al. [200]

### EBV

EBV, also known as HHV 4, is a member of the herpesvirus family that was originally isolated and detected in a human Burkitt’s lymphoma cell line [136]. EBV is now known to infect over 90% of the global population and remains as a symptomless life-long infection [136, 137]. Exposure to EBV occurs mostly in childhood or young adulthood. EBV has two life cycles in humans: an acute lytic cycle and a latent cycle. Production of new virions occurs in the acute lytic cycle, while the EBV remains undetectable in the host in the latent cycle. Latent EBV genomes are maintained in episomes in the nuclei of memory B cells and may also be detected in a proportion of T and natural killer cell subsets, as well as epithelial cells [138–140].

Dubbed as the first human oncovirus, the most well-known EBV oncoprotein is the latent membrane protein 1 (LMP1), a known direct target gene of Epstein–Barr virus EBV nuclear antigen 2 [141]. EBV LMP1 activates cellular signalling pathways associated with cancer development [142]. One such pathway is the epithelial-mesenchymal transition of a latently infected normal cell towards a cancer cell. Activation of this signalling pathway enhances epithelial cell migration and invasion that eventually contributes to metastasis [143]. Furthermore, it promotes cancer cell growth, survival and angiogenesis through the induction of growth factors, anti-apoptotic proteins and pro-angiogenic factors respectively. LMP1 can also be released in high levels within extracellular vesicles (EVs). LMP1 modifies the content of EVs, reprogramming recipient cells to increase their adhesion, proliferation and migration. Through these mechanisms, LMP1-containing EVs modify the microenvironment by promoting a permissive niche for tumourigenesis or metastasis [144]. The primary receptor for EBV, complement receptor 2, is expressed on astrocytes. Following viral entry, astrocytes exhibit increased proliferation [145]. The association between EBV and various CNS conditions, such as encephalitis and demyelinating diseases, alongside its frequent occurrence in CNS lymphomas, has sparked investigation into its potential involvement in gliomagenesis [146, 147] with inconsistent findings, indicating an absence of a definitive role for EBV in GB.

While EBV infection is high in the human population, the proportion of GB tumour tissues found to be positive for EBV DNA is lower than for JCV. Fonesca et al. detected 1/11 (9.1%) fresh frozen primary GB biopsy specimens to be positive for EBV DNA using PCR. The authors further sequenced the amplified EBV gene sequences and found that it was well matched with known published EBV genome sequences with a similarity rate of 95.5%, suggesting that EBV virus was present in these samples despite the low positivity in the cohort [148]. In a separate study by Lin et al., they screened for EBV DNA in 19 FFPE, 20 optimal cutting temperature (OCT) tissues, six fresh frozen GB tissues, as well as 32 fresh frozen and 17 FFPE non-neurological disease control brain specimens using multiplex droplet digital PCR. They detected the EBV *LMP-1* sequence in 4/45 (8.9%) of GB samples but not in any controls. All four samples that were found to be EBV positive were FFPE samples [149]. Stojnik et al. detected EBV *EBNA* sequence in only 3/33 (9.1%) samples that were made of 9 tumour biopsies and 24 surgical tumour resection samples from GB patients using PCR. Interestingly, all the patients were found to be seronegative for EBV antibodies and this suggested latent infection of EBV in these patients [150]. The highest proportion of EBV positive samples was in a study by Limam et al. who utilised multiple techniques simultaneously. They analysed 82 GB FFPE tissues by PCR, IHC and *in situ* hybridisation (ISH) by amplifying the EBV BamM region, EBV LMP-1 sequence and detecting EBER respectively. All PCR, IHC and ISH were performed in parallel with positive and negative controls. Using EBV specific PCR, EBV DNA was detected in 24/82 (29.3%) tissues. Consequently, IHC and ISH revealed 4/24 (16.7%) of the EBV DNA positive tissues displayed staining of EBV LMP1 and expression of EBER in tumour cells, respectively [151].

There were also studies being conducted on the publicly available sequencing datasets from The Cancer Genome Atlas (TCGA). In a comprehensive study by Strong et al., next-generation sequencing datasets from 157 primary GB and 5 normal brains, as well as whole genome sequencing (WGS) datasets from 51 primary GB samples. No virus was detected in 157 primary GB or 5 normal brain RNA-seq datasets, while low levels of EBV DNA were detected in 9/51 (18%) primary GB, 6/9 (66.7%) matched blood and 4/20 (20%) normal blood samples. All identified EBV DNA reads were in low abundance, ranging from 1-39 reads in primary GB samples and 1–5 reads in normal blood samples. The authors further noted that a true EBV association would result in samples with thousands of viral reads by DNA-seq [152–154]. On the contrary, Khoury et al. found no evidence of transcribed EBV viral elements in the total RNA database of GB tissue samples from TCGA [155]. Hashida et al. also failed to detect EBV in tumours from Japanese GB patients using real-time PCR analysis of the *LMP1* gene [156]. While Cimino et al. detected EBV DNA in 3/19 (15.8%) GB FFPE samples using next-generation sequencing (NGS), no EBV RNA transcript was found by RNA ISH. This suggests that the EBV may be in a latent cycle however, we cannot eliminate that other EBV transcripts might be expressed as the authors only examined one non-coding EBER RNA [157].

### HCMV

HCMV, otherwise also known as HHV 5, is a common virus in the herpesvirus family that infects most individuals at some point in their lives. HCMV infection is frequently asymptomatic, but it can cause severe illness in immunocompromised people [158]. HCMV has the ability to remain latent for life, but becomes reactivated in an immunosuppression and/or inflammation setting [159]. HCMV may favour tumour progression without being an oncogenic virus, which may explain the frequent presence of HCMV in tumour tissues [160]. IE1-72 and IE2-86 are two immediate-early (IE) proteins encoded by HCMV. They are known to activate expression of multiple genes simultaneously, and has been linked to dysregulatory events that can lead to the development of diseases such as cancer [161]. The HCMV UL55 gene encodes for glycoprotein B (gB), which plays a crucial role in multiple steps of HCMV pathogenesis, including cell penetration, cell-to-cell transmission and immune response activation [162]. pp65, the dominant protein found in the viral tegument, is highly abundant and serves as a primary component of extracellular virus particles [163]. pp65 is known to play a crucial role in evading and modulating the immune response of the host cell during HCMV infections [164]. The HCMV genome contains several oncogenes [165]. Research has shown that HCMV infection activates significant signalling pathways associated with cancers [166]. The IE-1 protein plays a crucial function in both initiating acute infections and reactivating the virus from latency. Research indicates that the IE1 gene is active in 90% of GB tumours [167] and facilitates oncogenic effects by interacting with p53 and other tumour suppressor proteins [168]. Another pivotal protein encoded in the HCMV genome, IE-2, exhibits oncogenic properties and induces cancer progression by influencing p53 and cell cycle advancement [169]. It is hypothesised that numerous other crucial genes contribute to enhancing the infectious potency of HCMV [165].

While the role of HCMV in pathogenesis has yet to be determined, treatment targeting the infection of HCMV in GB has improved their prognosis. Direct application of antiviral drug Valganciclovir alongside with the standard treatment of GB patients has demonstrated an improvement of median OS in newly diagnosed patients [170]. Alternate anti-viral approach has also been explored by stimulating dendritic cells with lysates from GB tissues resulted in the proliferation of HCMV-specific T cells in GB patients. Furthermore, CMV pp65-specific T cells have demonstrated the ability to eliminate autologous GB cells in vitro, implying the presence of HCMV epitopes in GB tumours [171, 172].

One of the first papers to associate HCMV with GB was carried out by Cobbs et al., who performed IHC with a monoclonal antibody specific for HCMV protein IE1-72 and pp65 and ISH to detect HCMV DNA on 22 GB FFPE tissues, 9 CNS diseases and 5 normal brains. The authors reported that HCMV IE1–72 was detected in all (100%) tumour cells but not in samples from CNS diseases and normal brain samples. In GB samples, the IE1-72 staining was located in the nucleus and perinuclear cytoplasm [167]. The presence of HCMV DNA was confirmed in the HCMV IE1-72 protein positive samples and pp65 staining was identified in two of the GB samples that revealed particles whose morphology was consistent with HCMV virions. Similar proportions of samples have tested positive for HCMV in subsequent studies [173–179]. The most detected marker of HCMV infection was pp65 followed by IE1-72, with most studies looking for two viral targets simultaneously. It is interesting to note that the percentage of HCMV positivity is higher in tumour tissue as compared to matched blood samples [177, 179].

A later study by Mitchell et al. showed 42/45 (93.3%) GB samples were positive for IE1-72 by IHC. To further confirm specificity, 33 of 45 samples were tested for pp65 reactivity. Of which, 30/33 (91%) samples were positive for pp65 by IHC. The authors also randomly selected 16 samples that showed to be HCMV positive via IHC. 16/16 (100%) samples were positive for HCMV DNA ISH. The authors also investigated the detection of HCMV in the peripheral blood of GB patients. 16/20 (80%) samples detected viral DNA in whole blood, while 0/17 (0%) of the healthy controls were positive for HCMV DNA [180]. In the same year, Scheurer et al. detected IE1-72 protein in 21/21 (100%) FFPE GB tissues. The authors also noted that IE1-72 was found in the perinuclear cytoplasm and in the nuclei of GB tumour cells. Importantly, the expression of IE1-72 protein and HCMV DNA were detected in the same tissue [173]. While Lucas et al. detected 8/49 (16.3%) were positive for IE1-72 and 25/49 (51%) were positive for pp65 in a cohort of 49 FFPE tumour tissues, these differences suggest variability of HCMV infection in GB [174].

Fonseca et al. inspected the prevalence of HCMV DNA in fresh frozen GB samples by performing PCR for pp65, with non-glial brain tumours as controls. All of the non-glial control specimens were found to be negative for HCMV, while 6/18 (33.3%) of the GB samples were HCMV positive, as defined by the presence of pp65 [175].

Ahani et al. investigated 16 GB frozen tumour samples, along with four non-tumour brain samples from trauma and epilepsy tissue as controls. Real-time (RT) PCR was carried out on cDNA from good quality total RNA that was extracted from tissues. HCMV DNA was detected in 12/16 (75%) GB tissues, while none were detected in normal tissues [176].

Santos et al. obtained 22 fresh tumours specimens and 20 peripheral blood samples from GB patients. DNA extraction and PCR were performed to determine the presence of HCMV. Authors performed quantitative PCR (qPCR) to detect the presence of pp65, while nPCR was used to detect gB. qPCR revealed 21/22 (95.5%) and 9/20 (45%) of samples were positive for the presence of pp65 in tumour and blood samples respectively. While nPCR detected 20/22 (91%) and 11/20 (30.1%) of samples were positive for gB in tumour and blood samples respectively. The authors correlated viral presence with samples obtained from the same patient. 9/19 (47.4%) and 11/18 (61.1%) of samples were positive for pp65 and gB in both tumour and blood specimens respectively [177]

Ding et al. examined the existence and role of HCMV components in GB. The authors analysed FFPE tissues from 19 GB specimens and 6 control brains from epilepsy patients by IHC and nPCR. HCMV IE1-72 was found to be expressed in 16/19 (84.2%) GB samples but none in the control brains, which were further assessed for HCMV related protein expression by detecting pp65 in 15/16 (93.8%) GB samples, while control tissues were pp65 negative. The authors further validated their IHC results by extracting total DNA and performing nPCR. HCMV UL55 gene was detected in 12/19 (63.1%) and 0/6 (0%) were detected in GB and control specimens respectively. Additionally, the authors analysed peripheral blood from GB patients and controls to detect of HCMV DNA utilising nPCR. Their findings revealed the presence of HCMV DNA in 7/19 (36.8%) GB patients, while no significant correlation was found between HCMV components in GB tissues and the presence of HCMV DNA in peripheral blood. Notably, none of the six controls with primary epilepsy had detectable HCMV DNA in their peripheral blood [178].

Bahador et al. employed both DNA and protein-based assays to identify HCMV in patient biopsies and corresponding blood samples. PCR was initially used to detect IE1 and the late gene UL83 (encodes pp65) in patient samples. UL83 was present in 26/159 (16.4%) of tumours but only in 12/119 (10.1%) of the matching blood samples. On the other hand, IE1 DNA was detected in 65/172 (37.8%) of tumours and only in 18/130 (13.8%) of patient blood samples [179].

Overall, despite the many positive outcomes detected, there were three studies that did not detect any HCMV in GB tissues or cell lines using a combination of molecular techniques such as IHC, PCR and ISH [181–183]. A more recent study by Tang et al. employed advanced deep-coverage whole-genome sequencing and detected HCMV in only 1/34 (2.9%) GB tumours, with only 2 CMV-mapping reads out of 1.30 billion in total from this single sample. Further examination revealed that both reads came from the CMV promoter, which makes up only 0.3% of the CMV genome and is commonly found in expression vectors that may inadvertently contaminate TCGA sequence libraries. The authors therefore believe there was no reliable evidence of CMV infection in any of the GB tissues [184].

### HHV6

HHV6 is a herpesvirus can that be categorised into two distinct variants: HHV6A and HHV6B [185]. Although both species replicate in T lymphocytes, they differ in entry receptor usage [186]. The distinction between the viruses is justified by the distinctive restriction endonuclease cleavage sites, growth patterns [187] and monoclonal antibody reactions [188]. HHV6A utilises CD46, a ubiquitous complement regulatory protein, while HHV6B mostly utilises CD134, a molecule only expressed on activated T cells [189].

Like other herpesviruses, HHV6 exhibits broad cellular tropism, although it replicates most efficiently in CD4 positive T cells. HHV6, like other human oncogenic herpesviruses, establishes latency in the lymphocytes and retains a strong immunomodulatory ability that can initiate both chronic inflammatory as well as immunosuppressive pathways [190]. HHV6 infection is usually cleared by the immune system with minimal issues, but it can reactivate in immunosuppressed patients and cause CNS dysfunction. HHV6 has been shown to integrate randomly into different chromosomes of somatic cells and gametes, habitually taking place in the telomere region. This integration mechanism is unique amongst human herpesviruses and allows the viral genome to be maintained during latency. Recent work provides evidence that the integrated HHV6 genome can be mobilised from the host chromosome, resulting in the onset of disease [191].

Chan et al. investigated the presence of HHV6 in 18 GB FFPE tumours using PCR, finding 1/18 (5.6%) of GB tissues were positive for HHV6 DNA [192]. Using a more sensitive and targeted detection method of nPCR, Cuomo et al. reported the detection of 14/31 (45.2%) positive HHV6 DNA in GB frozen tumours using nPCR. Of the 14 samples, 13 (92.9%) were positive for HHV6A while the remaining 1 was positive for HHV6B. 10/31 (32.3%) of the normal brain specimens were positive [193]. A similar trend was observed in a study by Chi et al. They detected HHV6 nucleic acids in 7/14 (50%) of GB tissues, while only 1/13 (7.7%) of normal brain tissues were positive. A follow up experiment on the HHV6 positive tumour samples using IHC revealed 5/14 (35.7%) were positive for HHV6 cytoplasmic and nuclear staining. No HHV6 immunoreactivity was detected in normal brain samples. The authors suggested that HHV6 infection and reactivation was a common feature in GB patients [194]. In a small cohort study by Lin et al., they showed that 3/19 (15.8%) FFPE GB samples and 3/20 (15%) frozen GB samples were positive for HHV6B, while only 2/49 (4.1%) of the non-neurological diseased control brain tissues were positive [149].

### VZV

VZV is an α-herpesvirus that causes chickenpox [195], a common childhood illness. A VZV infection results in chickenpox by infecting the respiratory mucosa, progressing into viremia where the virus is transported to and replicates in the skin. After acute infection, VZV establishes a life-long dormancy in the dorsal root ganglia of the host. Viral reactivation occurs in roughly 10-20% of VZV-infected individuals, resulting in shingles and/or other neurological conditions such as myelitis, post-herpetic neuralgia and encephalitis [196, 197].

Wrensch et al. suggested that their GB cohort has significantly lower levels of anti-VZV immunoglobulin G (IgG) than controls [198]. Another study performed by Wrensch et al. evaluated associations of IgG antibodies to VZV alongside other herpesviruses among GB patients and controls in blood. 109/115 (94.8%) of GB subjects tested positive for VZV IgG. No significant differences were established between the sample groups for seropositivity with other herpesviruses analysed in this study. The authors also implied that there was an inverse association between VZV immunity and GB [199]. Sjöström et al. analysed pre-diagnostic IgG levels for VZV along with HCMV, EBV and adenovirus using plasma samples from 61 GB patients. IgG antibodies for VZV, CMV and adenovirus were analysed by ELISA. Like Wrensch et al., lower levels of VZV-specific IgG were detected in GB cases compared to controls. The authors also reported no further associations between GB and antibody levels for EBV, HCMV, or adenoviruses. They also noted that since these plasma samples were collected pre-diagnosis, antibody levels were not impacted by tumour treatments [200].

While all of the viruses mentioned above were reported to be associated with an increased risk of GB, exposure to VZV has been shown to be an exception as it veers towards a lower risk of glioma [198, 199]. This trend has been consistently observed across multiple studies with varying VZV exposure assessment methods, such as total anti-VZV immunoglobulin G (IgG) levels [198–201] and self-reported history of chickenpox [199, 201, 202].

### Herpesvirus coinfection studies

Stojnik et al. reported HHV6 in 2/33 (0.06%), herpes simplex virus 2 (HSV2) in 1/33 (0.03%) and human enteroviruses (hEV) in 1/33 (0.03%) GB tumour samples tested. However, viral copy numbers for all viruses detected in GB tissue samples were generally very low. Once again, like EBV, none of the patients were seropositive for HHV6 or HSV2 antibodies in serum samples [150]. Wrensch et al. reported the highest proportion of GB patients with a viral presence in GB tissue. They reported 46/57 (80.7%), 45/57 (78.9%), 37/57 (64.9%) and 48/57 (84.2%) of their GB cohort were indeed seropositive for VZV, HSV, HCMV and EBV respectively by utilising serological IgG antibody binding ELISA assays. The authors further noted that GB samples were somewhat less likely than controls to have antibodies to VZV and EBV, but more likely than controls to have antibodies to HSV and HCMV [198]. Zavala-Vega et al. performed a retrospective study using brain tissue from 21 GB adult patients. EBV infection was detected by IHC by probing for LMP1 and EBER expression by ISH in 6/21 (28.6%) of patients. The authors also noted that mixed infections of EBV and CMV were detected in 5/21 (23.8%), whereas EBV and HSV-1/2 were noted in 4/21 (19%) patient samples. As this was a retrospective study based on paraffin-embedded tissue samples, the biggest limitation was the inability to detect IgG and IgM antibody levels [203].

The mounting evidence of herpesvirus presence, as presented earlier, is shedding light on the complex association between herpesviruses and GB. Ongoing research in this field holds the potential to unveil novel therapeutic avenues, bringing us closer to more effective strategies for combating GB.

## Papillomavirus

HPV is an epitheliotropic virus belonging to the papillomavirus family, that is known to cause 90% of all cervical cancer cases as well as anal, penile and oropharyngeal cancers [58]. HPV usually initiates cancer through the activity of the early 6 (E6) and 7 (E7) gene products. HPV E6 binds to and degrades the tumour suppressor protein p53, while E7 binds to and interferes with the protein-protein interaction of another tumour suppressor, Rb protein. Both p53 and Rb protein regulate the cell cycle, as disruption to these proteins allow continuous cell proliferation [204]. Despite HPV being primarily recognised as an epitheliotropic pathogen, studies have shown that virions can attach to cells originating from various tissues and species [205]. Furthermore, astrocytes express heparan sulphate proteoglycans, presumed to be the initial binding molecules for numerous HPV types [206]. The potential role of these molecules in facilitating HPV access to GB-initiating cells necessitates further investigation. All studies examining the role of HPV in GB are summarised in Table 3.

**Table 3:** Papillomavirus.

Year	Sample	Assay	Viral target type	Outcome	Reference
2014	52 FFPE tumour tissue	nPCR, chromogenic in situ hybridisation and IHC	DNA (L1 gene), Protein (L1 protein)	12 samples were positive for HPV, with 9 positive for HPV-6 and 3 positive for HPV-16. All HPV positive samples were positive for L1 protein	Vidone et al. [207]
2015	31 FFPE and 8 frozen GB tumours	qPCR, IHC	DNA (HPV16, E6 gene; HPV18, L1 gene), Protein (HPV L1 protein)	8/39 GB tumours were positive for HPV DNA, out of which 6 were positive for HPV-16 and 2 were positive for HPV-18 Only 3 HPV positive samples were used in IHC and they are all positive for L1 protein	Hashida et al. [156]

Hashida et al. detected the presence of HPV16 and HPV18 in 8/39 (21%) of the GB tissues in a Japanese cohort [156]. Similar findings were observed by Vidone et al. in an Italian cohort of GB patients where HPV DNA was detected in 12/52 (23.1%) by nPCR. Of the 12 HPV positive specimens, three samples were infected with HPV16, while the remaining 9 were found to be infected by the low risk HPV6 [207].

While functional investigations into the role of HPV in the initiation of GB were outside the scope of this preliminary analysis, we hypothesise that the virus might act as a contributing factor in gliomagenesis. Additional genetic alterations are likely necessary for HPV-related tumorigenesis, potentially involving the activation of oncogenes.

## Discrepancies between studies

Conflicting findings may occur because of the sensitivity of PCR and/or in-situ techniques utilised, or the small sample sizes. For example, despite the plethora of results presented above Cosset et al. analysed 20 GB biopsies and matched patient serum, where available, by semi-qPCR for the presence of the following common neurotropic viruses: HCMV, EBV, HHV6, VZV, HSV-1, HSV2, JCV, parechovirus (PeV), enterovirus (EV) and measles virus (MeV). None of the above-mentioned viruses were detected in any GB sample [208]. The inconsistency between these findings may be attributed by differences in brain tissue specimen preservation, experimental methodology and DNA detection methods. Most studies used IHC or PCR, focusing on the molecular identification of viral proteins or viral DNA, respectively. Understanding IHC can be complex due to background stains, such as formalin precipitates, hemosiderin, hematoidin and/or unspecific staining [209]. Off-target amplification during PCR can lead to false positives and lack of amplification of a specific amplicon cannot be taken as the absence of a viral genome [210]. To detect viruses, most studies focus on either a protein or a genomic sequence targeting technique, but not both. Some studies suggest that oncoviruses may trigger viral DNA recombinogenic activities that promote oncogenesis with the loss of viral genome in tumour cells [211]. Aside from differences in methodologies, there are many reasons that may explain the discrepancies observed between studies. These include differences in population or geography, the intrinsic heterogeneity of gliomas, individual genetic differences, variances in the targeted viral genes and the precision and sensitivity of the employed methods [53, 212, 213].

## Virotherapy and GB

Due to their host cell specificity and infection efficacy, viruses have also been explored as therapeutic agents for GB [214, 215]. This therapy is known as virotherapy and may have the ability to transform an immunosuppressive microenvironment of ‘cold tumours’ into immune-responsive ‘hot tumours’ [216, 217]. Virotherapy uses either oncolytic virus or oncolytic viral vectors to selectively induce apoptosis, necrosis and autophagy [218]. Subsequently, this leads to a release of tumour-associated antigens, viral pathogen-associated molecular patterns, damage-associated molecular patterns and various cytokines [219]. Additionally, oncolytic viruses can facilitate the activity of antigen-presenting cells, prompting their migration to the lymph nodes to stimulate cytotoxic CD8+T lymphocytes and attract them to the infection site, resulting in the killing of tumour cells [220].

Presently, over 30 oncolytic viruses have undergone clinical trials for the treatment of GB, with most being neurotropic retroviruses [221] and adenoviruses [222] capable of infecting neurons and glial cells, such as herpes simplex virus-1 (HSV) [223]. Adeno-associated viruses have demonstrated recent potential in preclinical trials for gene therapy targeting gliomas, although they have not yet undergone evaluation in clinical trials [224–226]. A comprehensive list of ongoing and completed clinical trials utilising diverse vectors for gene therapy in the treatment of GB has been explored by others (Caffery, 2019; Xu, 2021; Vecchio, 2019) [227–229]. There are some promising candidates that include adenovirus poliovirus (PVS-RIPO and DNX-2401) and retroviral vector (Toca 511) [230]. Notably, Toca 511 has demonstrated a lasting response in 21.7% of patients with malignant glioma, earning its expedited review status from the US Food and Drug Administration [231].

Despite extensive research on virotherapy, their impact has been limited, leading to only marginal improvements in OS and no approval from the US Food and Drug Administration for treating patients with GB. The major challenges are blood-brain barrier permeability, balancing antiviral and antitumour immunity and the sustainability of therapeutic effects. Nevertheless, some clinical successes with viral vectors have been observed in other types of cancers [227].

## Clinical trials using antivirals for the treatment of GB

Treating GB patients with antivirals is an emerging treatment for several reasons. Firstly, oncologists are constantly seeking new therapeutic strategies for GB due to the poor survival rates associated with currently approved treatments [232]. Secondly, oncolytic viruses have shown potential as an effective alternative approach for cancer treatment, including GB [230]. Antiviral drugs, such as Valcyte (valganciclovir), have been found to extend survival in GB patients, as demonstrated in a *New England Journal of Medicine* study [233]. These findings highlight the potential of antivirals to improve outcomes for GB patients and have contributed to the emergence of antivirals as a treatment option. All the clinical studies examining the potential of combining antivirals as part of GB treatment are summarised in Table 4. ClinicalTrials.gov was assessed on 31st October 2023, using the following key words: ‘Glioblastoma’, ‘Glioblastoma multiform’, ‘antiviral agents’ and ‘antivirals’ to identify any trials utilising antivirals as adjuvant therapy in GB patients. Nine clinical trials were identified to be using antiviral treatment in combination to standard of care. Of those antivirals, there were three different drugs: Valganciclovir (five trials), Nelfinavir (three trials) and combination of Ritonavir and Lopinavir (one trial).

**Table 4:** Clinical trials exploring antivirals as part of GB treatment.

Antiviral	Start date, Status, Cohort size	Primary purpose, Phase	Treatment schedule	Outcome (description)	ClinicalTrials.gov identifier (NCT number)
Valganciclovir	2006–2008, Completed with results, *n* = 42	Treatment, Proof of concept, randomised, double blind, placebo-controlled	1) Active comparator: two valganciclovir 450 mg tablets was orally administrated twice daily for 3 weeks before being reduced to one tablet twice daily until week 24 in addition to standard of care treatment. 2) Placebo comparator: two placebo 450 mg tablets were orally administrated twice daily for 3 weeks before being reduced to one tablet twice daily until week 24 in addition to standard of care treatment.	There were no significant differences observed in tumour volumes and median overall survival was similar in both groups. Interestingly, further analysis revealed a longer overall survival of 24.1 months in patients receiving more than 6 months of Valganciclovir when compared to 13.1 months in patients receiving Valganciclovir for 0 or less than 6 months and 13.7 months in contemporary controls. Overall survival at 4 years was 27.3% in patients receiving Valganciclovir for more than 6 months patients versus 5.9% in controls (*p* = 0.0466).	NCT00400322 Stragliotto et al. [236]
Valganciclovir	2005–2011, Completed with results, *n* = 15	Treatment, Phase 1b	Arm A: Patients with newly diagnosed unresectable malignant glioma to receive one of the three different doses of AdV-tk (3 × 10^10^, 1 × 10^11^, or 3 × 10^11^ vector particles) through stereotactic injection into tumour at time of biopsy. After 1–3 days, valacyclovir 2000 mg was administrated orally three times daily for 14. At day 9 after AdV-tk injection, radiation therapy was 2 Gy per day for 6 weeks to a total of 55 to 60 Gy. Standard dosage of TMZ was administered after completing valacyclovir treatment. 1) Arm B: Patients with newly diagnosed resectable malignant glioma to receive one of the three different doses of AdV-tk (3 × 10^10^, 1 × 10^11^, or 3 × 10^11^ vector particles) through stereotactic injection into tumour at time of biopsy. After 1–3 days, valacyclovir 2000 mg was administrated orally three times daily for 14. At day 9 after AdV-tk injection, radiation therapy was 2 Gy per day for 6 weeks to a total of 55–60 Gy. Standard dosage of TMZ was administered after completing valacyclovir treatment.	12/13 patients completed therapy, three at dose levels 1 and 2 and six at dose level 3 of AdV-tk. 2- and 3-year survival was 33% and 25% respectively. Patient-reported stable or improved quality of life after treatment. A significant CD3 positive T cell infiltrate was found in 4/4 (100%) tumours analysed after treatment. Three patients with MGMT unmethylated GB has a survival rate of 6.5, 8.7 and 46.4 months. There were no significant toxicities observed.	NCT00751270 Chiocca et al. [237]
Valganciclovir	2008–2017, Completed with results, *n* = 52	Treatment, Phase 2	Single arm, extension from NCT00751270: single dose of AdV-tk at 3 × 10^11^ vector particles delivered to tumour bed after resection on Day 0. Single course of valacyclovir at dose of 2 g orally three times per day for 14 days, after day 1–3 from AdV-tk injection. Standard radiotherapy started on an average of 7 days after AdV-injection.	Median overall survival was 17.1 months for combination therapy when compared to 13.5 months for radiotherapy only (*p* = 0.0417). Survival outcome for combinatory treatment at 1, 2 and 3 years was reported to be 67%, 35%, and 19% as compared to radiotherapy only at 57%, 22% and 8%. Patients with gross total resection were noted to have the highest benefit as reflected by their median OS of 25 months when compared to radiotherapy at 16.9 months (*p* = 0.0492); Survival outcome this group of patients at 1, 2 and 3 years was reported to be 90%, 53%, and 32% as compared to radiotherapy only at 64%, 28%, and 6%. There were no dose limiting toxicities; fever, fatigue and headache were the most common GMCI-related symptoms.	NCT00589875 Wheeler et al. [238]
Valganciclovir	2018–2023, Active but not recruiting, *n* = 36	Treatment, Phase 1, non-randomised single group assignment	Experimental cohort 1, MGMT unmethylated patients: injection of AdV-tk into wall of resection cavity. After 1–3 days, valacyclovir was administrated orally three times per day for 14 days. Patients will undergo five sessions of radiotherapy after an average of 8 days post-surgery. TMZ 75 mg/m^2^ provided at day 15 after completion of valganciclovir until MGMT was tested to be unmethylated. Nivolumab was given at day 15 post-surgery at 240 mg intravenously, every 2 weeks, 26 doses up to 52 weeks. Experimental cohort 2, MGMT methylated patients: injection of AdV-tk into wall of resection cavity. After 1–3 days, valacyclovir was administrated orally three times per day for 14 days. Patients will undergo five sessions of radiotherapy after an average of 8 days post-surgery. TMZ 75 mg/m^2^ was provided at day 15 after completion of valacycovir until MGMT was tested to be unmethylated. Nivolumab was given at day 15 post-surgery at 240 mg intravenously, every 2 weeks, 26 doses up to 52 weeks.	Not available	NCT03576612
Valganciclovir	2019-ongoing, Recruiting, *n* = 220	Treatment, Phase 2, randomised double-blinded study	All patients will be receiving standard of care concomitant treatment with TMZ 120 mg and radiotherapy 60 Gy. Active comparator arm: two Valganciclovir 450 mg tablets will be administrated orally twice daily for 6 weeks. After, the dosage will be reduced to once daily for the remaining 22.5 months. Placebo arm: two placebo tablets will be administrated orally twice daily for 6 weeks. After, the dosage will be reduced to once daily for the remaining 22.5 months.	Not available	NCT04116411
Nelfinavir	2009–2013, Completed, *n* = 6	Treatment, Phase 1, Single group assignment	Nelfinavir was administered 1 week before chemotherapy up till the end of chemotherapy. All patients start off with 1000 mg of nelfinavir twice a day in phase 1. If no does limiting toxicity was observed, the maximum dose of nelfinavir was 1250 mg twice a day.	Not available	NCT00694837
Nelfinavir	2009–2017, Completed, *n* = 21	Treatment, Phase 1, non-randomised single group assignment	Two doses were tested: 625 mg and 1250 mg twice a day for 6 patients. At maximum tolerated dose of nelfinavir, 15 additional patients would be enroled to evaluate further toxicity. Nelfinavir was given in combination with standard dose of temozolomide and radiotherapy.	The results showed that at 625 mg twice a day cohort was well-tolerated with patients, while in the 1250 mg cohort four patients were identifiesssd with dose limiting toxicity. Three of which were hepatotoxicity related and one gastrointestinal making the dose limiting toxicity rate of 22%. 2/18 patients had other serious adverse events for which dose were discontinued. The clinical trial had a median OS of 13.7 months and a median of 7.2 months of progression-free survival	NCT01020292 Alonso-Basanta et al. [241]
Nelfinavir	2009–2015, Terminated, *n* = 15	Treatment, Phase 1, single group assignment	7–10 days prior to commencing chemoradiotherapy, patients received oral nelfinavir mesylate twice a day and continue until the end of chemoradiotherapy. Patients also undergo radiotherapy once daily, 5 days a week while having oral temozolomide once daily for 6 weeks concurrently. 4 weeks upon completion of chemoradiotherapy and nelfinavir mesylate, patients were administered with only oral temozolomide once daily for 5 consecutive days. Treatment with temozolomide repeats every 28 days for up to six cycles in the absence of unacceptable toxicity or disease progression. Upon successful completion of study, patients were followed up regularly.	Terminated due to insufficient accrual.	NCT00915694
Combination of Ritonavir/ Lopinavir	2009-2011, Terminated, *n* = 19	Treatment, Phase 2, single group assignment	Patients were administered oral ritonavir and lopinavir twice a day in the absence of disease progression or unacceptable toxicity.	Not available	NCT01095094

### Valganciclovir

Valganciclovir is an oral anti-viral drug approved by the US Food and Drug Administration to treat a number of HSV infections, including CMV [234]. After consumption, valganciclovir is converted to its active antiviral component, acyclovir. Once absorbed by a herpesvirus infected cell, the acyclovir is converted to the active antiviral form, acyclovir triphosphate, by virally encoded and cellular enzymes. Subsequently, the acyclovir triphosphate inhibits the herpesvirus DNA polymerase and terminates viral DNA chain elongation, leading to inhibition of viral DNA replication [235].

An exploratory clinical trial examined the potential of including valganciclovir as an add-on to the standard of care therapy for patients with GB. There was no observed significant difference in tumour size and median OS between the treatment and placebo group. However, patients receiving more than 6 months of Valganciclovir had achieved a longer OS (24.1 months) when compared to patients receiving 0–6 months of Valganciclovir (13.1 months) and controls (13.7 months) [236]. In an extended retrospective study in a different group, 50 patients received valganciclovir in addition to standard therapy. Patients who received more than 6 months of Valganciclovir had achieved 90% survival rate at 2 years, with a median OS of 56.4 months [233].

At the same time another group was examining the inclusion of valganciclovir as part of a new treatment regime in a non-randomised, single group assignment Phase 1b clinical trial, NCT00751270. In this trial the safety and feasibility of delivering an adenoviral vector containing HSV thymidine kinase gene and valganciclovir to either patient with unresectable malignant glioma or resectable malignant glioma was examined. 33% of the cohort survived after 2 years and 25% after 3 years [237]. Given the positive trend, a portion of patients were enroled into a single group assignment phase 2a trial, NCT00589875. This was conducted across four different institutions where the patients were treated with a single dose of 3 × 10^11^ vector particles followed by radiotherapy. Combination therapy showed no dose limiting toxicities, with the most common side effects being fever, fatigue and/or headache [238]. This AdV-tk and valacyclovir combination is now explored in a phase 1 clinical trial as an add-on to standard of care treatment in patients with either MGMT unmethylated or methylated GB, NCT03576612.

The inclusion of valganciclovir as part of the standard of care to patients with GB is also being explored in another phase 2 randomised double-blinded study, NCT04116411, at the Karolinska University Hospital/Karolinska Institute. This is a multicentre trial that is currently recruiting participants and is anticipated to conclude in 2024.

### Nelfinavir

Nelfinavir is a nonpeptidic protease inhibitor that binds to the catalytic site of the human immunodeficiency virus (HIV) protease, thus preventing the cleavage of viral polyprotein precursors into mature, functional proteins that are essential for viral replication. Nelfinavir was approved for use in the United States in 1997 for the treatment of HIV infection in both adults and children [239]. Nelfinavir has shown promise as an anti-cancer agent [240]. As little as only two clinical trials completed investigating the use of nelfinavir and all of them administered the compound orally prior to radio- and chemotherapy.

A phase 1, non-randomised single group assignment study, NCT01020292 investigated dose limiting toxicity at two doses (625 mg and 1250 mg) in combination with standard temozolomide dose and radiotherapy. The results showed that at 625 mg twice a day cohort was well-tolerated with patients, while in the 1250 mg cohort four patients were identified with dose limiting toxicity. Three of which were hepatotoxicity related and one gastrointestinal making the dose limiting toxicity rate of 22%. 2/18 patients had other serious adverse events for which dose were discontinued. The study had a median OS of 13.7 months and a median of 7.2 months of progression-free survival [241].

NCT00694837 and NCT00915694, were both Phase 1, single group assignment clinical trial that explored administering nelfinavir prior to chemoradiotherapy to evaluate dose related toxicities. NCT00694837 was completed in 2013, but no information or results were posted. NCT00915694 was terminated due to insufficient accrual.

### Ritonavir and lopinavir

Both Ritonavir and Lopinavir are protease inhibitors used for treatment of HIV. Ritonavir binds to HIV-1 protease, which causes cleavage of protein precursors that generate new viral particles. Protease inhibitors interrupt this cleavage process, disrupting the production of new viral particles [242].

Lopinavir is an HIV-1 protease inhibitor, which is combined with ritonavir to increase its plasma half-life [243]. The combination of lopinavir with ritonavir is commonly used to boost protease inhibitors during HIV infection treatment. Due to the low bioavailability of lopinavir, it must be administered together with ritonavir to inhibit viral replication with higher drug concentrations [244]. A now terminated Phase 2 clinical trial, NCT01095094, administered oral ritonavir and lopinavir twice a day in the absence of disease progression. This study was terminated as it did not meet its primary objective, which was to achieve progression-free survival (4/16 patients).

## Modelling disease pathways in gliomas

Employing reverse translational approaches in syngeneic murine models revealed that mice bearing tumours that were perinatally infected with murine CMV exhibited a more adverse outcome compared to their uninfected counterparts. This was attributed, at least in part, to the increased recruitment of pericytes and augmented angiogenesis within the tumour microenvironment. The administration of antiviral therapy to infected mice enhanced their survival by reducing platelet-derived growth factor expression and disrupting angiogenesis [245].

Another study by Price et al. examined the role of CMV in GB by utilising a genetic mouse model subjected to perinatal murine CMV infection, as well as introduce HCMV into neurosphere cultures. Notably, murine CMV-infected mice with gliomas exhibited shortened survival compared to controls, indicating a potential contribution of murine CMV to glioma aggressiveness. The murine CMV presence, initially concentrated in CD45+ lymphocytes with active viral replication and local inflammation, later showed a generalised reduction in the brain. Importantly, murine CMV infection led to increased phosphorylated STAT3 levels in neural stem cells, suggesting a potential mechanism for glioma modulation. Correspondingly, HCMV was found to elevate phosphorylated STAT3 and increase proliferation in patient-derived GB neurospheres, an effect reversed by a STAT3 inhibitor both in vitro and in vivo. These findings underscore a potential association between CMV infection and a STAT3-dependent regulatory role in glioma development and progression, providing insights into the complex interplay between viral infection and glioma pathogenesis [246].

## Summary

Despite the rapidly expanding and evolving literature on the potential influence of viruses in cancer development and treatment, this field still presents numerous challenges and unanswered questions. Establishing causality becomes challenging when the viruses under discussion are prevalent in large proportions of the human population. Furthermore, it is conceivable that the intra-tumoral immune microenvironment may create conditions favouring productive viral replication, leading to reactivation from latency.

Several concerns and challenges have surfaced, demanding the attention of investigators. A significant hurdle involves discrepancies observed across various studies concerning viruses associated with GB. These inconsistencies persist even when employing similar techniques and are likely exacerbated, by variations in the sensitivity and precision of the methodologies used. These conflicting outcomes could be attributed to various factors, including population, geographic location, anatomical location of the tumour and tumour heterogeneity. Furthermore, variations in the handling or preparation of samples, such as section thickness, fixation conditions and antibody dilution, coupled with challenges related to paraffin-embedded tumour samples, may have contributed to the noted disparities.

The majority of studies utilised IHC or PCR, concentrating on the molecular identification of viral proteins or viral DNA, respectively. Understanding IHC can be intricate due to background stains, including formalin precipitates, hemosiderin, hematoidin and/or nonspecific staining [209]. Off-target amplification in PCR can result in false positives and the absence of amplification of a specific amplicon cannot be conclusively interpreted as the absence of the viral genome [210]. In the pursuit of virus detection, most studies focus on either a protein or a genomic sequence targeting technique, but rarely both. Some studies propose that oncoviruses might induce viral DNA recombinogenic activities that promote oncogenesis, leading to the loss of the viral genome in tumour cells [211].

In lieu of these biased approaches, the sequencing of GB tumours using next-generation platforms could be employed to identify viral or non-human nucleic acid sequences. Despite the advancements in genomic technology, establishing the association of ubiquitous viruses with GB tumours proves to be challenging. Rather than being discouraging, the disparities in studies examining viral aetiologies of GB should serve as motivation for a more thorough exploration.

## Data Availability

Data sharing not applicable to this article as no datasets were generated or analysed during the current study.
